# High Prevalence of Human Papillomavirus Type 18 in Oral Potentially Malignant Disorders in Thailand

**DOI:** 10.31557/APJCP.2021.22.6.1875

**Published:** 2021-06

**Authors:** Nithi Kaewmaneenuan, Suree Lekawanvijit, Surawut Pongsiriwet, Vuttinun Chatupos, Anak Iamaroon

**Affiliations:** 1 *Department of Oral and Maxillofacial Surgery, Faculty of Dentistry, Chiang Mai University, Chiang Mai, Thailand. *; 2 *Department of Pathology, Faculty of Medicine, Chiang Mai University, Chiang Mai, Thailand. *; 3 *Department of Oral Biology and Diagnostic Sciences, Faculty of Dentistry, Chiang Mai University, Chiang Mai, Thailand. *; 4 *Excellence Center in Osteology Research and Training Center (ORTC), Chiang Mai University, Chiang Mai, Thailand. *

**Keywords:** High-risk human papillomavirus, HPV16, HPV18- oral leukoplakia, oral lichen planus

## Abstract

**Objectives:**

The main objectives of this study were to investigate the detection rate of high-risk human papillomavirus types 16 and 18 (high-risk HPV16/18) in oral potentially malignant disorders (OPMDs) including oral leukoplakia (OL) and oral lichen planus (OLP) in a Thai population and their associations with demographic, risk habits, and clinicopathologic features.

**Methods:**

Paraffin-embedded formalin-fixed specimens from 101 OL and 59 OLP patients with patients’ demographic, risk habits, and clinicopathologic data were collected. Conventional qualitative polymerase chain reaction was used to detect high-risk HPV16/18 DNA. Associations between high-risk HPV type 16/18 and demographic, clinicopathologic, risk factors (tobacco and alcohol uses) of OPMDs were analysed by Chi-square or Fisher’s exact test. The results with p value less than 0.05 were considered statistically significant.

**Results:**

HPV16/18 DNA was found in both OL and OLP groups with the detection rate of 19.8% and 18.6%, respectively. Approximately 90% of high-risk HPV were HPV18 subtype. Additionally, in OL group, high-risk HPV was found more frequently in patients with moderate/severe dysplasia than that in mild dysplasia. Interestingly, in OLP group, high-risk HPV was only detected in atrophic/ulcerative subtypes. None of risk factors was associated with high-risk HPV.

**Conclusions:**

Approximately 19% of OPMDs were HPV16/18-positive. HPV18 DNA was predominantly detected in both OL and OLP patients (90%). Additionally, the detection rate of high-risk HPV was higher in more severe dysplastic cases of OL and more clinically severe cases of OLP.

## Introduction

Oral potentially malignant disorders (OPMDs) are a group of oral soft tissue lesions that have the potential to transform into malignant lesions, particularly oral squamous cell carcinoma (OSCC) (Warnakulasuriya, 2018). OPMDs include a wide variety of oral conditions, for example leukoplakia, erythroplakia, lichen planus, discoid lupus erythematosus, and actinic cheilitis. Risk factors of OPMDs are mainly tobacco use and alcohol consumption. Recently, high-risk human papillomavirus, especially types 16 and 18 has been reported as an emerging risk of OPMDs (Porter et al., 2018). 

HPV is a small circular DNA virus, consisting of three genome regions: early region (E), late region (L), and long control region (LCR). Based on their oncogenic properties, HPVs are divided into two groups: high-risk HPVs and low-risk HPVs (de Villiers et al., 2004). High-risk HPV has a high potency to induce malignant transformation in mucosal epithelial cells. The most common high-risk HPV in oral mucosa is HPV16 (Prabhu and Wilson, 2013). The routes of transmission to the oral mucosa include oral sexual transmission, direct contact, and mother-to-child (D’Souza et al., 2009; Syrjänen, 2018). HPVs specifically infect squamous epithelial cells through microwounds of the mucosa and are eventually embedded in the basal cell layer of the epithelium (Campisi et al., 2007). Having integrated into the host chromosomes, HPVs replicate and migrate into the upper epithelial cell layers. Subsequently, HPV oncoproteins E6 and E7 interrupt host’s cell cycle regulation by E6 oncoprotein abrogates apoptotic regulating proteins, including p53, bak, and procaspase, while E7 oncoprotein interrupts cell cycle regulating protein, pRb. As a result, the infected cells with high-risk HPVs can evade apoptosis and become actively proliferating (Prabhu and Wilson, 2013).

Oral leukoplakia (OL) is defined as a white epithelial lesion that cannot be diagnosed as any other diseases (Warnakulasuriya, 2018). Previous studies have revealed that malignant transformation rates of OL are in a range between 0.13% and 34% (Anderson and Ishak, 2015; Watabe et al., 2016). Prominent risk factors for malignant transformation of OL comprise the size of lesions more than 200 mm2, nonhomogeneous leukoplakia, advanced age over 50 years, severe dysplasia, and lesions located on the tongue or floor of the mouth (Holmstrup et al., 2006; Warnakulasuriya and Ariyawardana, 2016; Speight et al., 2018). A wide range of the detection rates of high-risk HPV in OL across the globe is between 0%–60% (Sugiyama et al., 2003; Campisi et al., 2004; Khovidhunkit et al., 2008; Sikka and Sikka, 2014; Bhargava et al., 2016). 

Oral lichen planus (OLP) is a chronic immunologic disorder of an unclear etiology (Warnakulasuriya, 2018). A meta-analysis study revealed that 1.1% of OLP transform into malignancies (Aghbari et al., 2017). Possible risk factors for malignant transformation of OLP include tobacco use, alcohol consumption, and hepatitis C virus infection. Moreover, atrophic/ulcerative OLP may have greater potential for malignant progression than other subtypes (Gonzalez-Moles et al., 2008; Speight et al., 2018). High-risk HPV has previously been detected between 0% and 30% in OLP cases (Campisi et al., 2004; Khovidhunkit et al., 2008; Razavi et al., 2009; Szarka et al., 2009; Arirachakaran et al., 2013). These discrepant results may be due to differences of ethnics, geography, and patients’ sexual and risk behaviors and underlying diseases. 

Since high-risk HPV may play a pivotal role for malignant transformation in OPMDs and little is known about the association between high-risk HPV and OPMDs in Thailand, we, therefore, aimed to investigate the infection rate of high-risk HPV particularly subtypes 16 and 18 in OL and OLP and to determine the association between high-risk HPV and demographic and clinicopathologic data, and classic risk factors including tobacco use and alcohol consumption in a Thai population.

## Materials and Methods

*Ethical approval*

The present study was approved by the Human Experimental Committee, Faculty of Dentistry, Chiang Mai University (No. 31/2019).

*Specimen collection*

The formalin-fixed paraffin-embedded (FFPE) specimens were collected from the archive of Oral Pathology Laboratory, Faculty of Dentistry, Chiang Mai University between 2015–2019. The inclusion criteria were OL located in the oral cavity and histopathologically diagnosed as hyperkeratosis or epithelial dysplasia and OLP with a microscopic confirmation. The exclusion criteria were histopathological diagnosis as cancer in part of lesions and the specimens with inappropriate tissue quality. 

*Data collection*

In this study, the demographic, risk factors, and clinicopathologic data of patients were collected from surgical pathology laboratory records. The demographic data consisted of age at the time of diagnosis and gender of patients. The clinicopathologic data included clinical types of OL and OLP, histopathologic features of OL, and sites of lesions. 

The ages at diagnosis of patients were divided in two groups: less than 45 years and equal to or more than 45 years. The clinical classification of OL was divided into two groups: homogeneous and non-homogeneous (van der Waal, 2018). While the clinical classification of OLP were divided into two groups: non-atrophic/ulcerative and atrophic/ulcerative types (Warnakulasuriya, 2018). The histologic features of OL were classified into three groups: hyperkeratosis, mild, and moderate/severe epithelial dysplasia. Risk factors of OPMDs including tobacco use and alcohol consumption were also recorded. All specimens were evaluated by three experienced oral pathologists at the Faculty of Dentistry, Chiang Mai University, Thailand. The definitive diagnoses of OL and OLP are based on the clinicopathologic criteria described by (Neville et al., 2015). 

*DNA extraction*

Each specimen was serially sectioned with the total thickness of 5-50 µm, depending on the tissue surface areas of each specimen and placed in a sterile tube. The specimen was then deparaffinized in xylene and washed with absolute ethanol. Subsequently, the pellet was dried and followed by genomic DNA extraction, using the QIAamp^®^ DNA FFPE Tissue Kit (Qiagen GmbH, Hilden, Germany). The extracted DNA was evaluated for quantity (absorbance at 260 nm) and purity (260/280 ratio) by the NanoDropTM 2000 Spectrophotometer (Thermo Fisher Scientific, Wilmington, DE, USA) and stored at -20^o^C until further DNA analysis.

*Quality assessment of the extracted DNA*

To assess the quality of the extracted DNA, the PCR amplification of the housekeeping beta-actin (β-actin) gene was tested in all specimens. The PCR was performed in a 50 μl reaction volume containing 1× PCR buffer, 0.2 mM dNTP mixture (InvitrogenTM, Carlsbad, CA, USA), 1.5 mM MgCl2, 0.2 µM of each, 1 unit of PlatinumTM Taq DNA polymerase (InvitrogenTM, Life Technologies, São Paulo, Brazil) and 5 µl of DNA template. Initial denaturation was operated at 95°C for 7 min, then 40 cycles of 94°C for 30 s, 55°C for 30 s, 72°C for 30 s, and a final extension at 72°C for 7 min. Positive and negative controls were included in each run. The PCR products were electrophoresed on a 1.2% agarose gel. The images were recorded using the ChemiDocTM Touch Imaging System (Bio-Rad Laboratories, Hercules, CA, USA). The β-actin-positive specimens were then further tested for HPV types 16 and 18.

*HPV16 and 18 DNA detection by PCR*

The amplifications of HPV16 and 18 DNA were conducted using the forward and reverse primers specific to the E6 gene of HPV16 and 18 as described in a previously study (Sritippho et al., 2016). The PCR components were the same as those of the β-actin gene. The DNA extracted from CasKi and HeLa cell lines was used for positive controls for HPV16 and 18, respectively. Sterile water was used as a negative control. The PCR conditions started with initial denaturation at 95°C for 7 min, followed by 45 cycles of 94°C for 30 s, 55°C for 30 s, and 72°C for 30 s, and a final extension at 72°C for 7 min. The PCR protocols were validated using serially diluted DNA extracted from CasKi and HeLa cell lines and limit of detections were 22.9 copies/µl and 1,550 copies/µl for HPV16 and HPV18, respectively.

*Statistical analysis*

The statistical analysis was performed using the IBM^® ^SPSS^®^ Statistics version 23. The Chi-square test (or Fisher’s exact test, two sided) was used to analyze the association between the HPV infection status and the categorical variables including age group, gender, risk factors, clinical type of OL and OLP, histopathologic grade of OL, and site of OL and OLP. The results with p values less than 0.05 were considered statistically significant.

**Figure 1:**
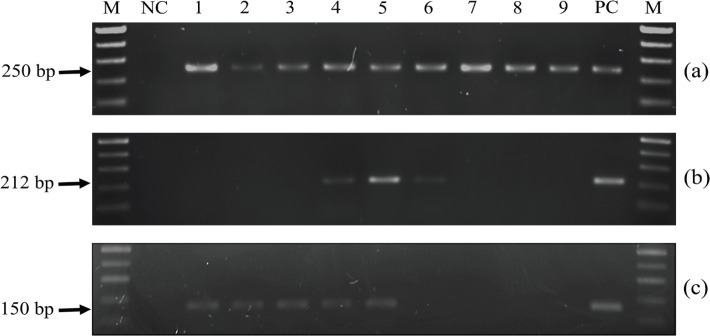
PCR Amplifications of Beta-Actin (a), HPV16 E6 (b), and HPV18 E6 (c) Genes in Specimens. Lane M: 100-bp DNA ladder marker; lane NC: negative control; lanes 1-3: HPV16-negative, HPV18-positive specimens; lanes 4 and 5: HPV16 and 18-positive specimens; lanes 6: HPV16-positive, HPV18-negative specimen; lanes 7-9: HPV16 and 18-negative specimens; lane PC: positive control

**Figure 2.**
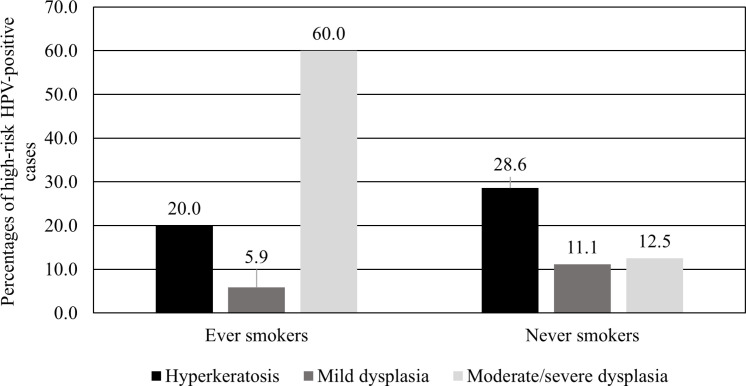
Percentages of High-Risk HPV-Positive Cases of Hyperkeratosis, Mild Dysplasia, and Moderate/Severe Dysplasia in Ever and Never Smoking OL Patients are Shown. Total patients in ever smokers group include hyperkeratosis (n=25), mild dysplasia (n=17), moderate/severe dysplasia (n=10). Patients in never smokers group include hyperkeratosis (n=14), mild dysplasia (n=9), moderate/severe dysplasia (n=8)

**Table 1:** Demographic, Clinicopathologic, and Risk Habits Data and Associations with HPV16/18 DNA

Variables	OL group (n=101)				OLP group (n=59)			
	HR-HPV Negative (n=81)	HR-HPV Positive (n=20)	No. of cases	P value^a^	HR-HPV Negative (n=48)	HR-HPV Positive (n=11)	No. of cases	P value^b^
Age group								
< 45 years	8 (88.9%)	1 (11.1%)	9	0.684†	11 (84.6%)	2 (15.4%)	13	>0.999†
≥ 45 years	73 (79.3%)	19 (20.7%)	92		37 (80.4%)	9 (19.6%)	46	
Gender								
Male	42 (79.2%)	11 (20.8%)	53	0.801	11 (84.6%)	2 (15.4%)	13	>0.999†
Female	39 (81.3%)	9 (18.8%)	48		37 (80.4%)	9 (19.6%)	46	
Tobacco use								
Never	25 (80.6%)	6 (19.4%)	31	0.691‡	31 (81.6%)	7 (18.4%)	38	>0.999†, ‡
Ever	40 (76.9%)	12 (23.1%)	52		3 (75%)	1 (25%)	4	
Unknown	16 (88.9%)	2 (11.1%)	18		14 (82.4%)	3 (17.6%)	17	
Alcohol consumption								
Never	14 (87.5%)	2 (12.5%)	16	>0.999†, ‡	20 (74.1%)	7 (25.9%)	27	0.394†, ‡
Ever	22 (84.6%)	4 (15.4%)	26		11 (91.7%)	1 (8.3%)	12	
Unknown	45 (76.3%)	14 (23.7%)	59		17 (85%)	3 (15%)	20	
Clinical subtypes								
Homogeneous OL	55 (78.6%)	15 (21.4%)	70	0.538	N/A	N/A	N/A	
Non-homogeneous OL	26 (83.9%)	5 (16.1%)	31		N/A	N/A	N/A	
Non-A/U OLP	N/A	N/A	N/A		8 (100%)	0 (0%)	8	0.330†
A/U OLP	N/A	N/A	N/A		40 (78.4%)	11 (21.6%)	51	
Histopathologic feature								
Hyperkeratosis	31 (77.5%)	9 (22.5%)	40	0.582	N/A	N/A	N/A	
Epithelial dysplasia	50 (82.0%)	11 (18.0%)	61		N/A	N/A	N/A	
Degree of dysplasia								
Mild	30 (90.9%)	3 (9.1%)	33	0.049*	N/A	N/A	N/A	
Moderate/severe	20 (71.4%)	8 (28.6%)	28		N/A	N/A	N/A	
Site								
Buccal mucosa	19 (70.4%)	8 (29.6%)	27	0.867†	39 (83.0%)	8 (17.0%)	47	0.270†
Gum	20 (83.3%)	4 (16.7%)	24		1 (100%)	0 (0%)	1	
Tongue	15 (83.3%)	3 (16.7%)	18		1 (100%)	0 (0%)	1	
Hard palate	9 (90%)	1 (10%)	10		2 (100%)	0 (0%)	2	
Soft palate	11 (78.6%)	3 (21.4%)	14		N/A	N/A	N/A	
Retromolar area	N/A	N/A	N/A		3 (100%)	0 (0%)	3	
Vestibule	6 (85.7%)	1 (14.3%)	7		2 (40%)	3 (60%)	5	
Unknown	1 (100%)	0 (0%)	1		N/A	N/A	N/A	
Total	80 (80.2%)	21 (19.8%)	101		48 (81.4%)	11 (18.6%)	59	

OL, oral leukoplakia; OLP, oral lichen planus; HR-HPV, high-risk human papillomavirus; N/A, not applicable; ^a^P value in OL group only; ^b^P value in OLP group only; *, Statistically significant with p values less than 0.05; †, Fisher’s exact test; ‡, The cases with missing data were excluded

**Table 2:** High-Risk HPV Subtypes in OPMDs

	HPV16 Alone (n=1)	HPV18 Alone (n=28)	Both HPV16 and 18 (n=2)	Total (n=31)	P value
OL	0 (0%)	18 (90%)	2 (10%)	20	0.273†
OLP	1 (9.1%)	10 (90.9%)	0 (0%)	11	

HPV, human papillomavirus; OL, oral leukoplakia; OLP, oral lichen planus; †, Fisher’s exact test

## Results

This study included 101 patients with OL and 59 patients with OLP. The demographic, clinicopathologic, and risk habits data and associations with HPV16/18 DNA are shown in Table 1. The detection rates of high-risk HPV were found at 19.8% and 18.6% in OL and OLP groups, respectively. Of all high-risk HPV detected, HPV18 were found approximately 90% in both OL and OLP groups (Table 2). Coinfection of HPV16 and 18 was detected at the rate of 10% merely in OL. The PCR products of some specimens are shown in Figure 1. 

In OL group, HPV16/18 infection were more prevalent in moderate/severe dysplasia cases than those of mild dysplasia cases (Table 1). Moreover, OL patients who had a history of smoking with moderate/severe dysplasia have significantly higher HPV16/18 infection rate than those with hyperkeratosis and mild dysplasia (p=0.007, Fisher’s exact test) (Figure 2). In OLP group, HPV16/18 were detected in only the atrophic/ulcerative OLP subtypes. While other factors including the age group, gender, risk factors, clinical subtypes of lesions, and sites of lesions were not significantly associated with high-risk HPV in both OL and OLP groups (Table 1).

## Discussion

In this study, we found a relatively high detection rate of high-risk HPV in OPMDs: OL and OLP at 19.8% and 18.6%, respectively. These results are consistent with several studies that have detected high-risk HPV in OL between 12% and 17% (Ostwald et al., 2003; Campisi et al., 2004; Acay et al., 2008), and OLP between 16% and 26% (Campisi et al., 2004; Szarka et al., 2009; Sahebjamiee et al., 2015). Previous similar investigations in central Thailand, however, have reported very low prevalences of HPV DNA in OPMDs, 0% of the detection rates in 17 OL and 16 OLP patients (Khovidhunkit et al., 2008) and 2.7% in 37 OLP patients (Arirachakaran et al., 2013). These discrepant results may be due to several factors. Firstly, the sample size of our study is much larger than that of the studies in central Thailand, hence, increased chance of the detection. Secondly, risk behaviors of the population in Thailand have alarmingly altered from the past. These alterations include younger age of first sexual intercourse, increased number of lifetime sexual partners, and more acceptation of teenager’s premarital sex (Techasrivichien et al., 2016; Pinyopornpanish et al., 2017). The third factor is a low level of HPV protection awareness in Thai persons. A self-reported study in Thailand revealed that only 1.9% of 521 undergraduate students had a history of vaccination. (Chanprasertpinyo and Rerkswattavorn, 2020). The reasons to decline vaccination were two-fold: the expenses of the vaccine and the lack of awareness of HPV infection (Ratanasiripong Nop et al., 2018; Chanprasertpinyo and Rerkswattavorn, 2020).

Globally, HPV16 is the most common high-risk HPV detected in precancerous and cancerous anogenital and oral lesions, and oropharyngeal cancers (Clifford et al., 2003; Herrero et al., 2003; Smith et al., 2007; Lin et al., 2018). Additionally, only HPV 16 was also detected at 1% in the saliva of normal Thai young adults (Wimardhani et al., 2015). Interestingly, in our study we found HPV18 was the most prevalent high-risk HPV detected in OPMDs with the detection rate of 90%. These results are in line with the studies from Italy and India (Campisi et al., 2004; Mathew et al., 2011). Moreover, our recent multicenter study in OSCC in Thailand demonstrated that the detection rate of high-risk HPV in northern Thailand was more prominent than that in other three regions, and HPV18 was the most identified subtype (Komolmalai et al., 2020). These findings suggest that HPV prevalence and subtypes be specific to geographic and ethnics of the populations. Interestingly, in head and neck cancer, HPV18 is more associated with OSCC, while oropharyngeal carcinomas are more HPV16-positive, indicating that differences of the anatomical sites may also play a role in infectivity of different HPV subtypes (Kreimer et al., 2005). 

In OL group, high-risk HPV infection rate was increased in patients with more severe epithelial dysplasia. This result was consistent with a previous study in the Italian population that found a higher infection rate in patients with moderate and severe dysplasia (Angiero et al., 2010). The higher detection rate of high-risk HPV in moderate/severe dysplasia may suggest that high-risk HPV infection is more stabilized in more severe dysplastic lesions. Having integrated in the host’s genome, HPV DNA then transforms those dysplastic cells into malignant cells. That is why malignant transformation of the oral mucosa is more commonly found in moderately and severely dysplastic lesions (Neville et al., 2015). Moreover, our study reported the highest HPV infection rate in ever smokers with moderate/severe dysplasia compared to those with hyperkeratosis or mild dysplasia. These findings support that smoking and HPV could synergistically promote carcinogenesis (Aguayo et al., 2020). 

Interestingly, our study revealed that HPV infection was only found in atrophic/ulcerative OLP subtypes, which is consistent with previous meta-analysis that reported a strong association between HPV infection and atrophic/ulcerative OLP (Ma et al., 2016). These findings may reflect that atrophic/ulcerative OLP has no surface protection, hence HPV can easily transmit into the basal cells of the mucosal epithelium. In addition, immunosuppression of the lesions from long-term treatment of topical steroid with atrophic or ulcerated lesions may induce the environment of OLP for being susceptible to HPV infection (Syrjänen et al., 2011; Syrjänen, 2018). However, further studies are needed to elucidate the possibility that high-risk HPV can induce malignant transformation in atrophic and ulcerative OLP. 

Collectively, a relative high detection rate of high-risk HPV in OPMDs in our study suggests that dental personnel should be more aware that a subpopulation of OPMDs is high-risk HPV-positive and these lesions may further progress to malignancies, particularly in OL with moderate/severe dysplasia, especially patients who had a history of smoking, and in atrophic/ulcerative OLP. Since the main means for prevention of HPV infection is vaccination, we recommend that both boys and girls should be provided with HPV vaccine for preventing precancer and cancerous lesions of the anogenital organs and head and neck regions including the oral cavity. Previous studies have also indicated that HPV vaccination strategy is the most cost-effective way to decrease the incidence of cancers caused by high-risk HPV (Termrungruanglert et al., 2012; Aguilar et al., 2016). 

In conclusion, HPV18 infection was significantly predominant than HPV16 in OL and OLP. Interestingly, high-risk HPV infection rates were increased in more severely dysplastic oral lesions. Additionally, only atrophic and ulcerative OLP subtypes were infected by high-risk HPV in patients with OLP.

## Author Contribution Statement

Nithi Kaewmaneenuan: Conceptualization, Methodology, Validation, Formal analysis, Investigation, Resources, Data Curation, Writing-Original Draft, Visualization. Suree Lekwanavijit: Methodology, Validation, Resources, Writing-Review & Editing, Supervision. Surawut Pongsiriwet: Methodology, Validation, Resources, Writing-Review & Editing, Visualization, Supervision. Vuttinun Chatupos: Resources, Writing-Review & Editing, Supervision. Anak Iamaroon: Conceptualization, Methodology, Validation, Resources, Writing-Review & Editing, Visualization, Supervision, Project administration.
